# Von Willebrand factor and hematogenous cancer metastasis under flow

**DOI:** 10.3389/fcell.2024.1435718

**Published:** 2024-08-30

**Authors:** Wenxuan Xu, Xi Tan, Morgan L. Li, Hanzhi Xu, Jasmine Villegas, Hongxia Fu

**Affiliations:** ^1^ Division of Hematology and Oncology, Department of Medicine, University of Washington, Seattle, WA, United States; ^2^ Institute for Stem Cell and Regenerative Medicine, University of Washington, Seattle, WA, United States; ^3^ Department of Bioengineering, University of Washington, Seattle, WA, United States; ^4^ Bloodworks Research Institute, Seattle, WA, United States

**Keywords:** Von Willebrand factor, cancer, metastasis, flow, platelets, endothelial cells

## Abstract

Hematogenous metastasis involves cancer cell migration to different locations from the primary tumor through the blood circulation. Von Willebrand factor (VWF) has been shown to play an important role in tumor cell adhesion to and extravasation from the endothelial cell lining of blood vessel walls during cancer metastasis. VWF may contribute to this process by interacting with tumor cells, endothelial cells, and platelets through various cell membrane receptors, such as platelet glycoprotein (GP)Ibα, P-selectin, α_ν_β_3_ and α_IIb_β_3_ integrins, and glycocalyx. Blood flow can mechanically extend and activate VWF to bind platelets and associate intermolecularly with other VWF molecules in plasma or on the surface of endothelial cells, cancer cells, or platelets. This suggests a mechanoregulatory role of VWF in mediating the interactions between VWF and these cells to promote cancer cell adhesion to blood vessels. In this review, we will summarize the current knowledge of VWF function and the role of hydrodynamic forces in hematogenous cancer metastasis.

## 1 Introduction

Tumor metastasis is a main cause of cancer-related mortalities (Chaffer and Weinberg, 2011). Hematogenous metastasis is one of the major ways for cancer migration, in which cancer cells detach from the primary tumor, enter the bloodstream, travel to different locations through the blood, and extravasate from blood vessels to form a secondary tumor (Chambers et al., 2001). To extravasate from the bloodstream, these circulating tumor cells (CTC) may first adhere to endothelial cells (EC) in the inner liner of blood vessels, and then migrate through EC.

Many blood cells and proteins can contribute to hematogenous cancer metastasis, such as platelets, leukocytes, and von Willebrand factor (VWF) (Nash et al., 2002; Patmore et al., 2020). Elevated plasma VWF levels often appear in cancer patients, suggesting an important role of VWF in cancer development (Liu et al., 2014; Pepin et al., 2016; Patmore et al., 2020). VWF has been identified as an essential mediator to regulate CTC adhesion to and extravasation from the EC in blood vessel walls (Gadducci et al., 1994; Zietek et al., 1996; Eppert et al., 2005; Wang et al., 2005; Boccaccio and Medico, 2006; Terraube et al., 2007; Liu et al., 2014). In blood circulation, VWF always experiences hydrodynamic forces generated by flow and plays a mechanoregulatory role in recruiting platelets or other VWF molecules to the EC surface during thrombus formation in hemostasis and thrombosis (Fu et al., 2017; Fu et al., 2021). Such flow-induced interactions between VWF, platelets, and EC may also contribute to CTC adhesion and migration during metastasis.

In this review, we will focus on the evidence indicating VWF functions in hematogenous cancer metastasis. We will also elucidate the connection between the mechanoregulatory role of VWF and cancer metastasis in the presence of blood flow. Understanding how flow regulates VWF function in hematogenous metastasis may provide important insights into seeking efficient therapeutic interventions.

## 2 VWF structure, synthesis, and secretion

**FIGURE 1:**
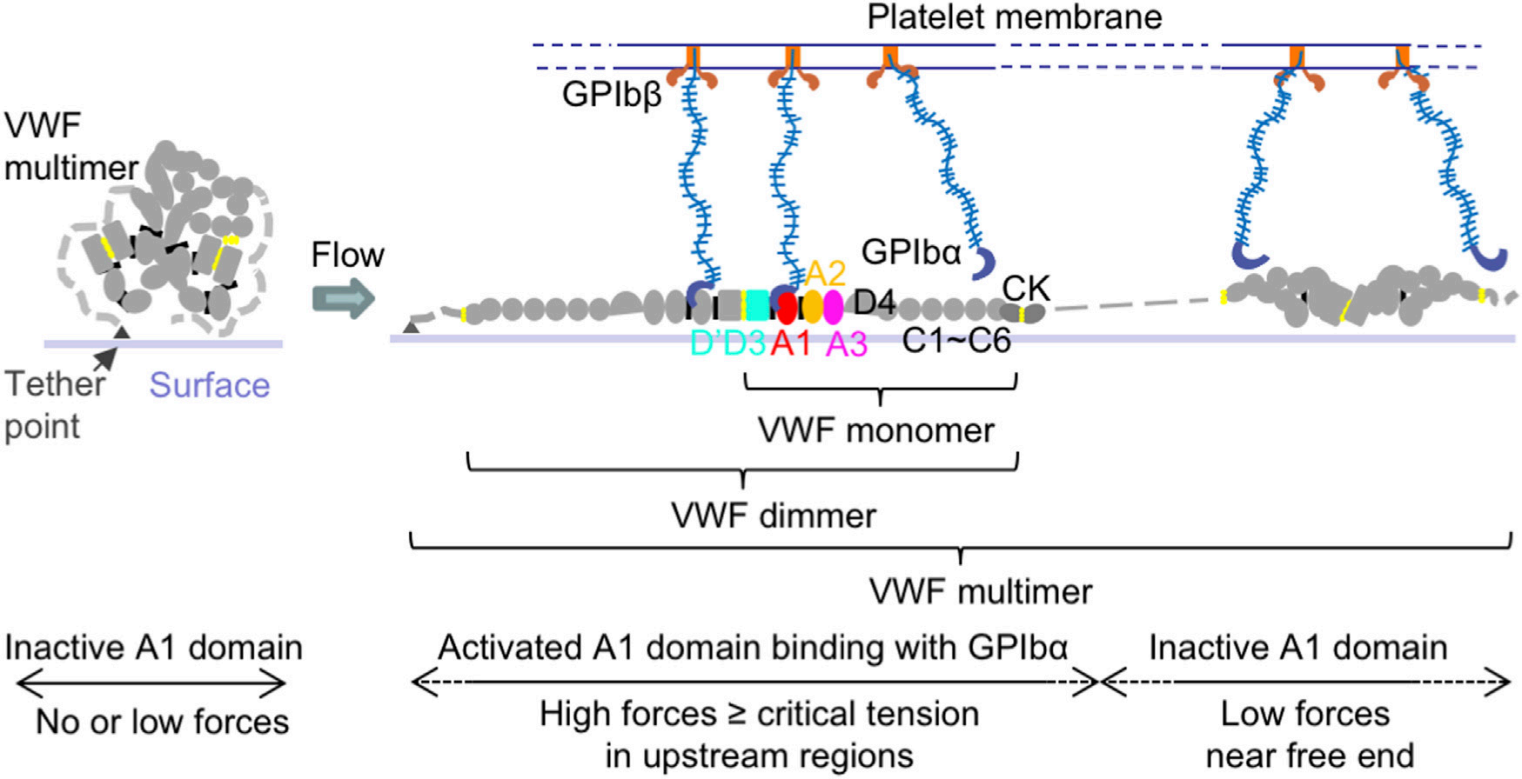
VWF structure and activation to bind platelet GPIbα. Schematic of VWF domain, monomer, dimer, multimer, and activation under flow. Within a surface-tethered VWF multimer exposed to flow, only the VWF monomers with tension reaching a critical value extend and become activated to bind GPIbα on the platelet surface, while other monomers with the force below the critical tension do not bind.

VWF is a multimeric blood protein consisting of covalently linked multi-domain monomers (Sadler, 1998). Each mature monomer contains 2,050 residues forming D’D3, A1, A2, A3, D4, C1-C6, and CK domains (Figure 1). Each multimer consists of variable numbers of such monomers. The multimeric structure of VWF allows multiple binding sites of one VWF molecule to interact with many different ligands from other cells or biomolecules, such as cancer cells, platelets, EC, and other VWF multimers, to have different functions at the same time.

Among VWF domains, the A1 domain is responsible to bind platelet glycoprotein (GP)Ibα and regulate platelet adhesion to vascular walls in primary hemostasis (Lankhof et al., 1995; Sadler, 1998; Savage et al., 1998; Sadler, 2005; Reininger, 2008; Kim et al., 2010; Blenner et al., 2014; Springer, 2014; Kim et al., 2015) (Figure 1). The VWF-A2 domain is the site for ADAMTS13 (a disintegrin and metalloproteinase with thrombospondin type 1 motif, member 13) enzyme cleavage, which decreases VWF multimer size and platelet binding (Zhang et al., 2009; Ying et al., 2010; Zhou et al., 2011; Xu and Springer, 2012). Through its D’D3 domains (Foster et al., 1987; Castro-Nunez et al., 2013; Shiltagh et al., 2014; Chiu et al., 2015; Yee et al., 2015; Dagil et al., 2019; Dong et al., 2019), VWF can also form a complex with blood coagulation protein factor VIII (FVIII) to protect FVIII from rapid clearance in the bloodstream, and thus play a role in blood fibrin clot formation in secondary hemostasis (Weiss et al., 1977; Gentry et al., 1981; Montgomery et al., 1982; Nesheim et al., 1988). The VWF-D’D3 domains are also potential binding sites for P-selectin, which is stored in and secreted from EC Weibel-Palade bodies (WPB) and platelet α-granules, to attach VWF on the EC surface (Padilla et al., 2004; Michaux et al., 2006). Both VWF-A1 and A3 domains can bind collagen (Hoylaerts et al., 1997; Nishida et al., 2003; Bonnefoy et al., 2006; Brondijk et al., 2012). The VWF-C module has Arg-Gly-Asp (RGD) motif specifically binding to integrin α_IIb_β_3_ and α_ν_β_3_ on the surface of EC, platelets, and cancer cells (Denis et al., 1993; Zhou et al., 2012).

In blood vessels, VWF is synthesized in two main sources, platelets and EC (Sadler, 1998; Springer, 2014). Synthesized VWF is stored in platelet α-granules and EC WPB, ready to be released upon the activation of platelets and EC, respectively. Recently, some cancer cells, such as gastric adenocarcinoma cells and osteosarcoma cells, have also been found to actively express VWF (Eppert et al., 2005; Mojiri et al., 2017; Yang et al., 2018).

Secreted VWF exists in two main forms in the bloodstream, circulating VWF in blood plasma and tethered VWF at the cell surface of EC, platelets, or cancer cells. Surface-tethered VWF multimers may interact with each other or provide a platform to attract circulating plasma VWF, a process known as intermolecular self-association (Savage et al., 2002; Shankaran et al., 2003; Ulrichts et al., 2005; Barg et al., 2007; Dayananda et al., 2010; Chung et al., 2016; Zhang et al., 2019; Fu et al., 2021). Through self-association, single VWF molecules can assemble into large VWF strings attached to the vessel wall, which increases the amount of tethered VWF available on the EC surface (Savage et al., 2002; Dayananda et al., 2010). Such large VWF strings can also increase the tensile force generated by blood flow and lower the hydrodynamic flow required to activate VWF function. On the other hand, tethering may induce VWF conformational changes to unfold the A2 domain and increase the probability of ADAMTS13 cleavage to reduce platelet binding (Zhang et al., 2009; Zhou et al., 2011; Xu and Springer, 2012). Tethered VWF can recruit platelets even on an intact platelet or EC surface under physiological blood flow (Andre et al., 2000; Dong et al., 2002). Therefore, tethered VWF may play an important role in cancer metastasis by providing a “bridge” for cancer cell adhesion to EC directly through interacting with cancer cells or indirectly through recruiting platelets to EC and facilitating cancer cell adherence to platelets (McCarty et al., 2000; Jain et al., 2007).

## 3 VWF function in hematogenous cancer metastasis

The primary roles of VWF are to regulate platelet adhesion and aggregation, as well as carry and protect FVIII from degradation in blood plasma for fibrin clot formation (Weiss et al., 1977; Montgomery et al., 1982; Sadler et al., 1987; Sadler, 1998; Savage et al., 1998; Sadler, 2005; Reininger, 2008; Springer, 2014). Besides these established roles of VWF in hemostasis and thrombosis, high VWF levels in cancer patients have been linked to tumor metastasis (Gadducci et al., 1994; Zietek et al., 1996; Eppert et al., 2005; Wang et al., 2005; Boccaccio and Medico, 2006; Terraube et al., 2007; Liu et al., 2014). Inhibition of VWF binding to platelet integrin α_IIb_β_3_ by antibodies reduced pulmonary cancer metastasis in a mouse model (Karpatkin et al., 1988). Blocking VWF binding to platelet GPIbα by antibodies was shown to suppress cancer metastasis (Qi et al., 2018).

Interestingly, opposite results have been found in VWF deficient mice, showing that the initial establishment of metastatic foci using B16-BL6 melanoma cells increased in VWF deficient mice comparing with the wild-type mice (Terraube et al., 2006; Terraube et al., 2007). Injection of purified VWF together with tumor cells could correct the phenotype and show similar metastatic foci in both wild-type and VWF deficient mice.

The controversy between the above enhancement and abatement roles of VWF in cancer metastasis was later explained by apoptosis induced by VWF in B16-BL6 melanoma cells, which involves VWF-tumor cell binding in an integrin-dependent manner (Terraube et al., 2006; Terraube et al., 2007). However, such an apoptosis function of VWF is not universal and can be depressed by a VWF cleavage protease, ADAM28 (a disintegrin and metalloprotease domain 28), secreted by many other types of tumor cells, suggesting that the role of VWF in cancer metastasis is not simple and may vary in different types of tumor cells (Mochizuki et al., 2012).

The enhancement role of VWF in cancer metastasis was proposed to involve several mechanisms, such as facilitating tumor cell adhesion to EC, increasing vascular permeability for cancer cell extravasation, stimulating angiogenesis to promote tumor growth, and supporting leukocyte recruitment (Patmore et al., 2020; Colonne et al., 2022). VWF has been shown to directly interact with leukocytes or recruit leukocytes through platelets, which may contribute to vascular permeability and angiogenesis (Patmore et al., 2020). However, roles of VWF to both increase and decrease vascular permeability have been reported, and both pro- and anti-angiogenic roles of VWF have also been revealed (Kawecki et al., 2017; Patmore et al., 2020). The mechanism to resolve these conflicts remains to be determined. Compared to these uncertainties, the role of VWF in assisting tumor cell adhesion on EC is well established by many studies. Here, we will focus on the contribution of VWF in interacting with platelets, EC, cancer cells, and several of their receptor proteins, such as platelet GPIbα, P-selectin, α_ν_β_3_ and α_IIb_β_3_ integrins, and glycocalyx, in cancer cell adhesion to the EC surface.

### 3.1 VWF assists platelet, EC, and cancer cell interactions

**FIGURE 2:**
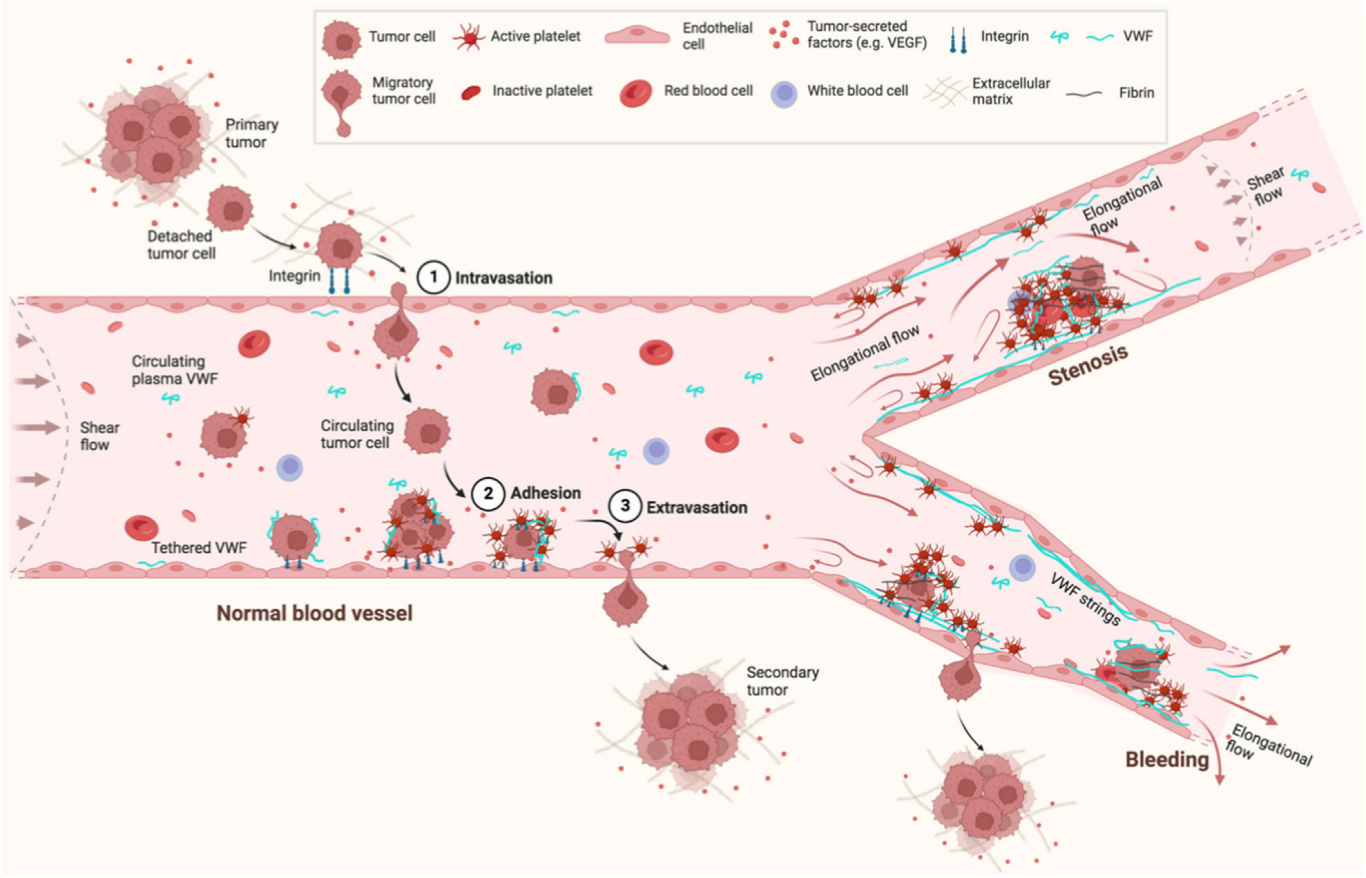
Schematic of VWF and hematogenous cancer metastasis under flow. Cancer cells detach from a primary tumor, enter a nearby blood vessel (1. Intravasation) and circulate in the bloodstream. Some circulating tumor cells eventually adhere to EC in the blood vessel wall (2. Adhesion) and extravasate from the EC (3. Extravasation) to form a secondary tumor. VWF is a mediator to regulate the interactions between cancer cells, EC, and platelets. VWF also provides a “bridge” to recruit cancer cells directly or through platelets to the EC surface. Flow can not only elongate and activate VWF to bind platelets or other VWF molecules to form VWF strings, but also mediate cancer adhesion and migration. Flow is changed from shear flow in normal blood vessels to elongational flow in blood vessels with junctions/branches, stenosis, or bleeding. This figure was created with BioRender.com.

During hematogenous metastasis, cancer cells can interact with platelets and EC on the blood vessel wall, eventually extravasate, and enter new destinations to colonize secondary sites (Gasic et al., 1968; Nash et al., 2002) (Figure 2). Such interactions between cancer cells, platelets, and EC have been proposed to not only protect clearance of tumor cells by the immune system, but also enhance the adhesion of both platelets and tumor cells to the vessel wall. In this process, platelets may play an assisting role in promoting cancer cell migration.

Under physiologic conditions, inactive platelets circulate in blood vessels but do not interact with EC on the vessel wall. Platelets may be activated by thrombin or adenosine diphosphate (ADP), which promotes interactions between platelets and tumor cells, and the adhesion of tumor cells on EC surface (Nash et al., 2002). Activated platelets may also secrete growth factors, such as vascular endothelial growth factor (VEGF), which promote angiogenesis and facilitate tumor cell proliferation. Cancer patients have significantly high concentrations of VEGF, which can induce megakaryocyte maturation and thus enhance turnover of platelets in these patients (Salven et al., 1999). VEGF can also stimulate EC to release the Tissue Factor, which may induce platelet adhesion to EC resulting in aggregation of tumor cells and platelets adhesion to EC (Verheul et al., 2000). Secretions from cancer cells (tumor-derived supernatant) can activate EC to secrete VWF, forming long strings attached on the EC surface, which can promote platelet and cancer cell adhesion with each other and on the EC surface (Goerge et al., 2007; Bauer et al., 2015; Dhami et al., 2022). Without ADAMTS13 to cleave long VWF strings, the number of metastatic foci can significantly increase in ADAMTS13-deficient mice (Goertz et al., 2016), suggesting these tethered VWF strings assist cancer cell adhesion on EC.

Besides EC and platelets, many tumor cells of non-endothelial cell origin, such as osteosarcoma, gastric adenocarcinoma, and gliomas, also have the ability to express and store VWF (Eppert et al., 2005; Mojiri et al., 2017; Yang et al., 2018). Such cancer cell-derived VWF has also been shown to promote the interaction of cancer cells with platelets and EC to enhance metastasis (Mojiri et al., 2017; Yang et al., 2018). Inhibition of cancer cell-derived VWF expression can thus reduce cancer cell metastasis (Liu et al., 2014; Pepin et al., 2016).

### 3.2 VWF binds receptors on platelets, EC, and cancer cells

Several receptors on platelets, EC, and cancer cells have been proposed to provide attachment points for VWF on these cells, in which the major ones are platelet GPIbα, P-selectin, α_ν_β_3_ and α_IIb_β_3_ integrins, and glycocalyx (Denis et al., 1993; Sadler, 1998; Padilla et al., 2004; Huang et al., 2009; Zhou et al., 2012; Kalagara et al., 2018).

VWF interacts with platelet membrane protein GPIbα via the VWF-A1 domain (Lankhof et al., 1995; Sadler, 1998; Savage et al., 1998; Sadler, 2005; Reininger, 2008; Springer, 2014). Such interaction can be formed through conformational transition of the VWF-A1 domain from inactive to active state induced by blood flow (Kim et al., 2010; Ju et al., 2013; Kim et al., 2015; Fu et al., 2017; Fu et al., 2021). Blocking VWF binding to platelet GPIbα can reduce not only cancer-associated thrombosis but also cancer cell metastasis (Jain et al., 2007).

P-selectin is expressed in EC, platelets, and many cancer cells such as in lung, ovarian, lymphoma, and breast cancer (Shamay et al., 2016). It can directly recruit cancer cells and mediate the interaction of activated platelets and cancer cells, and the adhesion of cancer cells to stimulated EC under blood flow (McCarty et al., 2000; Morganti et al., 2000; Chen and Geng, 2006). Although the mechanism regarding the interaction between VWF and P-selectin is unclear, P selectin has been shown to anchor on the EC surface and the VWF-D’D3 domains have also been proposed to be potential binding sites for P-selectin (Padilla et al., 2004; Michaux et al., 2006). Plasma P-selectin levels have been found elevated in many cancer patients (Castellon Rubio et al., 2020). Inhibition or removal of P-selectin reduced platelet-tumor cell interactions and attenuates cancer metastasis (Kim et al., 1998; Borsig et al., 2001; Borsig et al., 2002). P-selectin deficient mice also generated fewer cancer metastases compared to wild-type mice (Kim et al., 1998).

Integrins α_IIb_β_3_ and α_ν_β_3_ have been shown to interact with the RGD motif in the VWF-C module and contribute to cancer cell adhesion to the EC surface (Denis et al., 1993; Zhou et al., 2012). Platelet integrin α_IIb_β_3,_ also known as the glycoprotein GPIIb/IIIa, is capable of interacting with VWF, cancer cells and EC to facilitate cancer cell adhesion to platelets and EC (Felding-Habermann et al., 1996; Bombeli et al., 1998; McCarty et al., 2000). Integrin α_ν_β_3_ is expressed on EC and several malignancies, such as melanoma, glioma, ovarian and breast cancer, and mediates cancer cell adhesion to EC and also interaction with both VWF and platelets (Felding-Habermann et al., 1996; Bombeli et al., 1998; Felding-Habermann et al., 2001; Pilch et al., 2002; Terraube et al., 2006). Direct interactions between integrin α_IIb_β_3_ and α_ν_β_3_ can also mediate tumor cell, platelet, and EC interactions and facilitate hematogenous tumor metastasis (Lonsdorf et al., 2012).

Among VWF domains, the A1 domain is positively charged, while the others are all negatively charged. The VWF-A1 domain may be responsible to bind with negatively charged glycosaminoglycans in the glycocalyx on the EC surface by electrostatic interactions (De Ceunynck et al., 2013; Kalagara et al., 2018). Using VWF as a “bridge”, cancer cells and platelets may thus interact with EC through GPIbα, P-selectin, α_ν_β_3_ and α_IIb_β_3_ integrins, and/or glycocalyx to facilitate cancer cell adhesion to the EC surface.

## 4 Mechanoregulation of VWF in cancer metastasis

During hematogenous cancer metastasis, blood flow applies hydrodynamic forces on cells and biomolecules in the blood vessels, which can affect VWF assisted CTC adhesion to and extravasation from EC layer in blood vessel walls (Follain et al., 2018; Follain et al., 2020). A number of physical and mechanical parameters may affect this process, such as blood vessel dimension, flow pattern, and hydrodynamic force (Follain et al., 2020).

The diameters of human blood vessels vary across a wide range, e.g., from a small capillary (∼8 µm) to large artery (∼4,000 µm) or vein (∼5,000 µm) (Müller et al., 2008). CTC can possibly become entrapped in and adhere to capillaries due to size constraints. EC in the inner layer of arteries are generally thicker than those in veins. Capillaries have the thinnest vessel walls, only consist of a single layer of EC, which probably makes capillaries more eligible for cancer cell extravasation than arteries and veins.

Under physiological blood flow conditions, the average wall shear stress varies over a large range: 1–4 dyn cm^-2^ in venous vessels, 4–50 dyn cm^-2^ in arterial vessels, and 10–20 dyn cm^-2^ in capillaries (Arfors and Bergqvist, 1974; Arfors and Bergqvist, 1975; Papaioannou and Stefanadis, 2005; Pantos et al., 2007; Follain et al., 2020). During bleeding, shear stress can greatly increase. For example, shear stress can increase up to 12-fold from 50 to ∼600 dyn cm^-2^ due to raised flow rate (up to 6-fold in proximal arterioles (Arfors and Bergqvist, 1974)) and vasoconstriction (Arfors and Bergqvist, 1975). Blood flow pattern changes from shear to elongational flow at the junctions/branches of normal blood vessels, or in the vessels with stenosis or bleeding (Figure 2).

VWF has been shown to be a mechanoregulatory protein. VWF experiences tensile force generated by hydrodynamic flow in blood vessels, and many of VWF functions are regulated by tension. By stretching VWF multimers or fragments, single molecule studies have revealed that the characteristic activation tension to initiate VWF binding platelet GPIbα is at a range of ∼10–20 pN (Kim et al., 2010; Kim et al., 2015; Fu et al., 2017; Jiang et al., 2019; Arce et al., 2021). Under flow, VWF is activated through a two-step conformational transition: first, elongation from compact to linear form, and subsequently, a tension-dependent nanometer-length local transition to an active state for GPIbα binding (Fu et al., 2017) (Figure 1). Similar force range (∼10–20 pN) was also required for unfolding the VWF-A2 domain for ADAMTS13 cleavage (Zhang et al., 2009; Ying et al., 2010; Müller et al., 2016). Under physiological flow conditions, secretion from cancer cells and/or platelets can activate EC to quickly release VWF to form strings (Goerge et al., 2007; Dhami et al., 2022). We have shown that tension can induce VWF elongation from compact to linear form and then intermolecular self-association between VWF molecules when tension is above ∼11 pN (Fu et al., 2021). Such interactions between VWF multimers may happen when VWF secreted from EC, platelets, cancer cells, or within the same VWF multimer. Hydrodynamic forces have been shown to favor adhesion of tumor cells to the EC surface (Follain et al., 2018), which can also be regulated by the above tension-induced VWF recruiting platelets and other VWF molecules on the EC surface. After binding with platelets and cancer cells, VWF experiences higher forces applied by these cells under flow, which may in turn activate more monomers in VWF strings to recruit more platelets and cancer cells and promote cancer cell adhesion to the EC surface.

Both circulating plasma VWF and VWF attached to the cell surface can contribute to cancer adhesion on the EC surface by binding to platelets, EC, and cancer cells through membrane proteins, including platelet GPIbα, P-selectin, integrins, and glycocalyx (Denis et al., 1993; Sadler, 1998; Padilla et al., 2004; Huang et al., 2009; Zhou et al., 2012; Kalagara et al., 2018). The binding of VWF to platelet GPIbα requires elongation of VWF molecule at a force above characteristic activation tension (Fu et al., 2017). Surface immobilized VWF but not free VWF in solution can interact with integrin ανβ3 on the surface of EC or cancer cells under static conditions (Denis et al., 1993; Terraube et al., 2006). Immobilization may induce conformational changes in VWF. These experimental data suggest that conformational changes of VWF are required to initiate VWF binding to platelets, cancer cells, and EC. As described above, flow induced VWF-GPIbα binding has been widely studied. However, the role of flow induced VWF conformational changes in initiating VWF binding to P-selectin, integrins, and glycocalyx and mediating cancer cell adhesion to EC is still unclear.

Under normal blood flow, circulating VWF remains in a compact conformation, which could possibly prevent it from interacting with cancer cells. In the event of vascular injury or stenosis, flow rate is elevated and shear flow changes to elongational flow, in which the hydrodynamic forces applied on VWF also increase. Such hydrodynamic forces could possibly induce VWF extension and initiate its binding to cancer cells. Hydrodynamic forces could also induce intermolecular interaction (VWF self-association) between circulating VWF and surface attached VWF to promote binding of VWF with EC, platelets, and cancer cells.

Shear flow can be explained as a linear superposition of an elongational flow and a rotational flow (Smith et al., 1999). Compared to shear flow, elongational flow is predicted to extend VWF to a greater degree under the same flow rate (Sing and Alexander-Katz, 2010). Elongational flow usually exists at the sites of blood vessels where the dimensions change, such as the junctions/branches of normal blood vessels, or vessels with stenosis or bleeding (Figure 2). The majority of tumor cells being arrested at arterio-venous junctions has been observed in a zebrafish model (Follain et al., 2018), which may be due to the elongational flow induced VWF extension and activation to recruit platelets and tumor cells under physiological flow rates at these sites. Thus, VWF attached on a surface, especially in the cases of vascular injury or stenosis, potentially plays a more important role in cancer migration than circulating plasma VWF under the same flow condition.

## 5 Discussion

VWF has been shown to be an important mediator in cancer cell adhesion and extravasation during hematogenous metastasis. Regulating VWF function under flow can change its interactions with cancer cells, platelets, and EC, and also control cancer cell development. However, multiple challenges remain in fully elucidating the mechanisms of VWF function in this process. For example, how does VWF contribute to vascular permeability and angiogenesis for cancer cell extravasation from blood vessels? How do various flow rates and patterns mediate VWF interaction with cancer cell to regulate its rolling, arrest, and adhesion on the EC surface? Many roles of VWF in cancer have been reported, including cancer-associated thrombosis (CAT), cancer metastasis, cancer apoptosis, inflammation, vessel permeability, and angiogenesis (Liu et al., 2014; Pepin et al., 2016; Patmore et al., 2020; Khorana et al., 2022). How does flow contribute to the interplays between these roles? How do flow-induced platelet binding and VWF self-association between VWF secreted from EC, platelets, and tumor-derived VWF contribute to cancer adhesion and migration? VWF is the natural carrier for FVIII, which was found to be expressed and secreted by cancer cells (Walker et al., 2022). FVIII levels are also elevated in many cancer patients (Castellon Rubio et al., 2020). Injection of FVIII in hemophilic mice was found to enhance the lung cancer metastasis, suggesting an important role of FVIII in the blood-borne phase of metastatic cascade (Langer et al., 2006). However, it is unclear how VWF interacts with FVIII and contributes to the metastatic process in the presence of flow. Mechanosensing plays an important role in cancer cell adhesion and progression (Hu et al., 2017; Yao et al., 2022). How do hydrodynamic forces generated by various blood flow affect cancer cell mechanosensing, and how does VWF contribute to this process? Answering these questions will improve our understanding of VWF function in cancer metastasis as well as the contribution of fluid mechanics in these processes, which can potentially provide important insights into developing new anti-metastatic strategies to treat cancer.
